# A dynamic model of lignin biosynthesis in *Brachypodium distachyon*

**DOI:** 10.1186/s13068-018-1241-6

**Published:** 2018-09-19

**Authors:** Mojdeh Faraji, Luis L. Fonseca, Luis Escamilla-Treviño, Jaime Barros-Rios, Nancy L. Engle, Zamin K. Yang, Timothy J. Tschaplinski, Richard A. Dixon, Eberhard O. Voit

**Affiliations:** 1 The Wallace H. Coulter, Department of Biomedical Engineering, Georgia Institute of Technology and Emory University, 950 Atlantic Drive, Atlanta, GA 30332-2000 USA; 2 BioEnergy Sciences Center (BESC), Oak Ridge National Lab, Oak Ridge, TN USA; 3 BioDiscovery Institute and Department of Biological Sciences, University of North Texas, 1155 Union Circle #305220, Denton, TX 76203-5017 USA; 4 Oak Ridge National Laboratory, P.O. Box 2008, Oak Ridge, TN 37831 USA

**Keywords:** *Brachypodium distachyon*, *Medicago truncatula*, *Panicum virgatum*, Pathway analysis, *Populus trichocarpa*, Recalcitrance

## Abstract

**Background:**

Lignin is a crucial molecule for terrestrial plants, as it offers structural support and permits the transport of water over long distances. The hardness of lignin reduces plant digestibility by cattle and sheep; it also makes inedible plant materials recalcitrant toward the enzymatic fermentation of cellulose, which is a potentially valuable substrate for sustainable biofuels. Targeted attempts to change the amount or composition of lignin in relevant plant species have been hampered by the fact that the lignin biosynthetic pathway is difficult to understand, because it uses several enzymes for the same substrates, is regulated in an ill-characterized manner, may operate in different locations within cells, and contains metabolic channels, which the plant may use to funnel initial substrates into specific monolignols.

**Results:**

We propose a dynamic mathematical model that integrates various datasets and other information regarding the lignin pathway in *Brachypodium distachyon* and permits explanations for some counterintuitive observations. The model predicts the lignin composition and label distribution in a *BdPTAL* knockdown strain, with results that are quite similar to experimental data.

**Conclusion:**

Given the present scarcity of available data, the model resulting from our analysis is presumably not final. However, it offers proof of concept for how one may design integrative pathway models of this type, which are necessary tools for predicting the consequences of genomic or other alterations toward plants with lignin features that are more desirable than in their wild-type counterparts.

**Electronic supplementary material:**

The online version of this article (10.1186/s13068-018-1241-6) contains supplementary material, which is available to authorized users.

## Background

Lignin is the world’s second most abundant organic polymer after cellulose; it is estimated to constitute 30% of all organic carbon on earth. Lignin is essential for a plant’s structural stability and for its water transport. In sufficient quantities, lignin makes plant materials, such as stems and roots, very hard. As a consequence, it poses a substantial challenge in terms of animal feed digestibility, because its irregular aromatic structure is difficult to decompose enzymatically. Indeed, if the total amount of lignin in feed for cattle and sheep could be reduced, millions of dollars could be saved [1], and genetically engineered reduced lignin alfalfa has recently been commercialized [2, 3]. Lignin is similarly a grand challenge for the biofuel industry, because the heteropolymer is interwoven with cellulose and hemicellulose in plant cell walls, which impedes access of hydrolytic enzymes to these polysaccharides [4–6]. This so-called *recalcitrance* poses a critical obstacle to the US Department of Energy’s goal of replacing significant amounts of gasoline with ethanol [7], as biomass requires expensive pretreatment that drastically increases the cost of ethanol production. Given the potentially large returns on investment, recalcitrance has become the target of investigation in many research labs.

To address recalcitrance at its origin, a good understanding of lignin biosynthesis is obligatory. However, an intuitive understanding of this process is confounded by the grid-like structure of the pathway and the multiple use of the same enzymes for different substrates, which in combination lead to a complicated nonlinear flux distribution. For instance, it is difficult to predict how the pathway might respond to knockdowns of genes coding for enzymes that catalyze distinct reactions within the pathway. Further complicating these challenges is the observation that the lignin pathway is similar, but not exactly the same in structure and regulation across different plant species. It was shown in recent years that computational modeling can greatly assist in comprehending the functionality of the lignin pathway [8–12].

The first step of a computational metabolic pathway analysis is typically a stoichiometric representation that accounts in a binary manner for all known reactions leading from one or more precursors to the desired products. Such a stoichiometric model is very valuable, but not sufficient for the important second goals of explaining counterintuitive observations and of guiding experiments into targeted genetic alterations of the pathway toward particular biotechnological goals, such as favorable changes in lignin amount or composition. To achieve these types of goals, a dynamic model is needed that permits the evaluation of transitions from the original state of the pathway to the new, intended state, where the “state” is defined by metabolite concentrations and magnitudes of fluxes.

Previous computational studies demonstrated that mathematical modeling can assist with the quantitative characterization of structural and regulatory features of the lignin biosynthetic pathway. As a case in point, a counterintuitive observation in alfalfa demonstrated that knockdowns of genes early in the pathway, before the reactions toward S- and G-monolignols diverge, nevertheless lead to different proportions of S- and G-lignin. We ultimately resolved these discrepancies by developing stoichiometric and dynamic models for black cottonwood (*Populus trichocarpa*) [10], alfalfa (*Medicago sativa L.*) [8], and switchgrass (*Panicum virgatum*) [11]. These purely computational analyses led to the postulate of metabolic channels, whose functionality was subsequently validated in alfalfa [8].

**Fig. 1:**
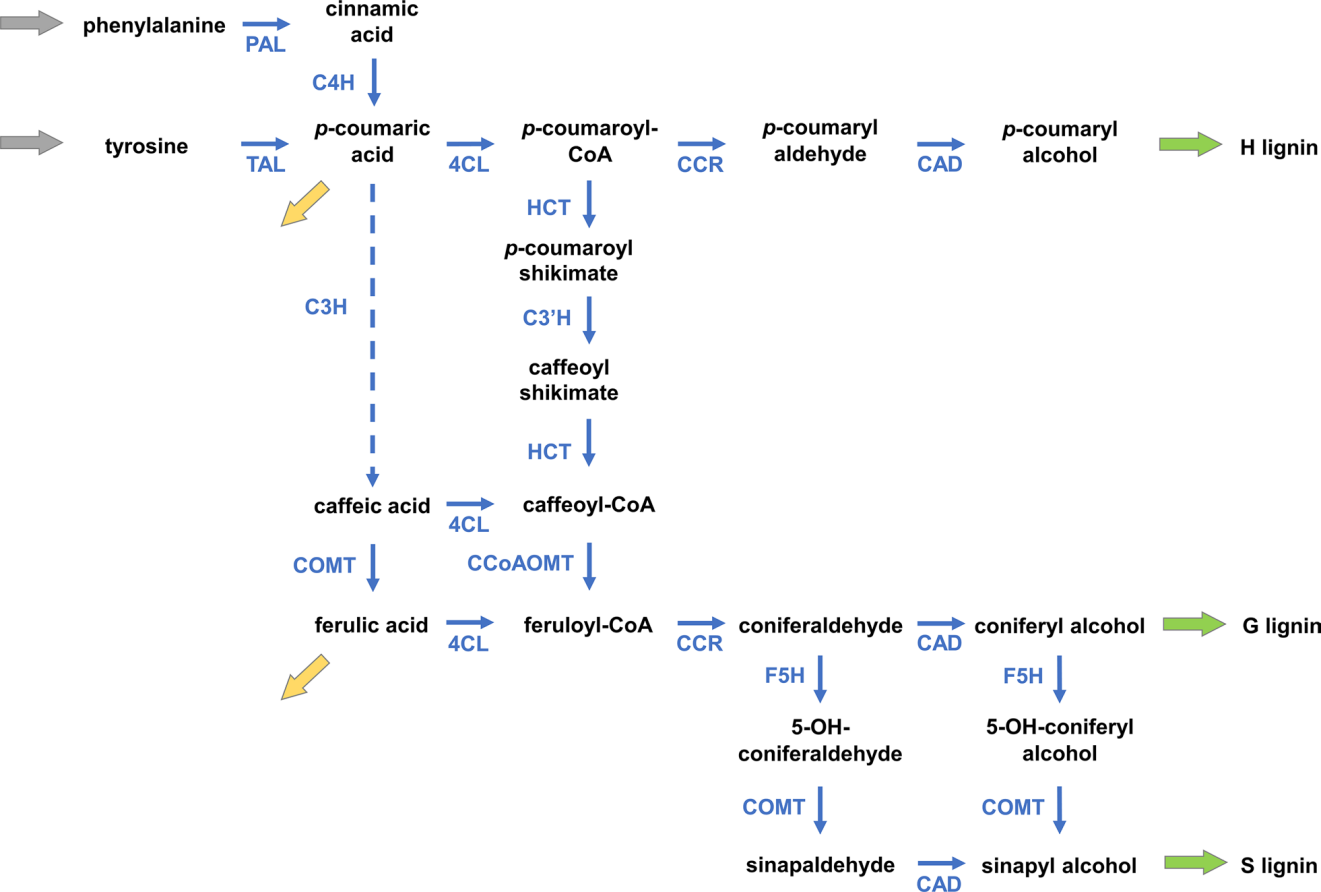
Putative lignin biosynthesis pathway in *Brachypodium distachyon*. Inputs from phenylalanine and tyrosine appear to merge early in the pathway, at the pool of *p*-coumaric acid. This early convergence renders the observation curious that feeding labeled phenylalanine or tyrosine results in distinctly different incorporations of label into different lignin monomers. The pathway through C3H (dashed) is tentative

Here we employ similar computational tools to investigate the pathway in the grass *Brachypodium distachyon*, which has become a model plant for lignin analyses in recent years. The modeling work is relevant for two specific reasons. The first is the need to explain a confusing observation resulting from labeled phenylalanine (Phe) and tyrosine (Tyr) feeding experiments. Namely, experimental data revealed a differential incorporation of either Phe or Tyr into the various lignin units [11], even though the two amino acids enter the lignin pathway at almost the same source metabolite and quickly lead to the same precursors for the remainder of the pathway (Fig. 1). Specifically, the experiments demonstrate that upon supplying labeled Phe, a higher incorporation of labeling is funneled into G-lignin, whereas a higher incorporation into S-lignin is observed for labeled Tyr feeding. Second, a future goal of this modeling effort is to make reliable predictions regarding the lignin amount and composition in the organism in response to single and double gene knockdowns.

To explain the differential responses to Phe or Tyr labeling, we started again with the strategy of stoichiometric modeling, using the putative structure of the pathway of lignin biosynthesis in *Brachypodium* (Fig. 1) as basis. While the resulting model represented much of the pathway appropriately, it turned out to be incapable of reproducing the observed differential S- and G-lignin production. In fact, intense further model exploration and analysis led to the conclusion that the assumed topology of the pathway is not consistent with distinct G and S preferential pathways from Phe and Tyr, respectively. This inability to match observations persisted even if we took into account metabolic channels, as proposed to be present in alfalfa and switchgrass [8, 11]. To resolve the discrepancy, we analyzed the pathway structure further and came to the following conclusions.

The paths initially starting from Phe and Tyr diverge into different effluxes at the *p*-coumaric acid branch point (Fig. 1), but the subsequent fluxes eventually merge at the feruloyl-CoA node thus leaving no direct opportunity for material to flow into a particular monolignol that would be specific to the initial source of Phe or Tyr. Exploring various biologically reasonable alternatives, the modeling analysis led to the conclusion that a single compartment is insufficient to explain the data and that it is necessary to take into account the spatial localization of the pathway enzymes. To assume different locations for enzymatic activity actually seems very reasonable, because experimental observations report that three of the pathway enzymes, namely C4H, C3′H and F5H, are bound to the outer surface of ER, whereas other enzymes are commonly assumed to be free in the cytosol. Accordingly, we developed a static model with two “compartments”, the cytosol and the outer ER surface, to examine the role of enzyme localization for the differential incorporation of Phe and Tyr into different monomer units. One should note that these compartments are not truly separated from each other but constitute localized centers of enzymatic activity with some material and information flow between them. A static two-compartment model of this type indeed allowed us to explain the labeling data [13].

While this result is reassuring, the steady-state model is not sufficient for the quantitative exploration of responses to alterations, such as gene knockdowns. Thus, if one were to ask which enzyme activities should be changed to alter the total amount or the composition of lignin in a targeted manner, the static model would not be capable of offering an answer. Instead, such an answer requires a dynamic model that represents a sufficiently wide operating range with sufficient accuracy. We develop such a dynamic model in the work described here.

A dynamic model is necessary for a variety of analyses, which include—but are not restricted to—time course simulations. Of particular interest for us is the predictability of responses to introduced gene modulations, which cannot be accomplished reliably with a pure steady-state metabolite model. For instance, a 50% decrease in the abundance of an enzyme does not necessarily correspond to a 50% decrease in the flux catalyzed by this enzyme, because such perturbations are often confounded by changes in metabolites (for a clear demonstration of such compensation in a different context see [14]). Moreover, the new steady state can usually not be calculated due to the fact that metabolic pathway systems are underdetermined and nonlinear. This is where the power of dynamic models comes to assist, as the dynamics of perturbations can be simulated conveniently and reliably, if the model is adequate. In particular, if stable solutions exist, the simulations and mathematical analyses will reveal them. Furthermore, the dynamic model permits optimization methods that predict particularly useful gene modifications, as we have shown in the context of lignin synthesis elsewhere [15].

## Results

### Review of features of the static model of lignin biosynthesis in *Brachypodium*

We described elsewhere the procedures for designing a stoichiometric model for the pathway of lignin biosynthesis in *Brachypodium* [13] and it suffices here to review the main features, which are important for the following.

**Fig. 2:**
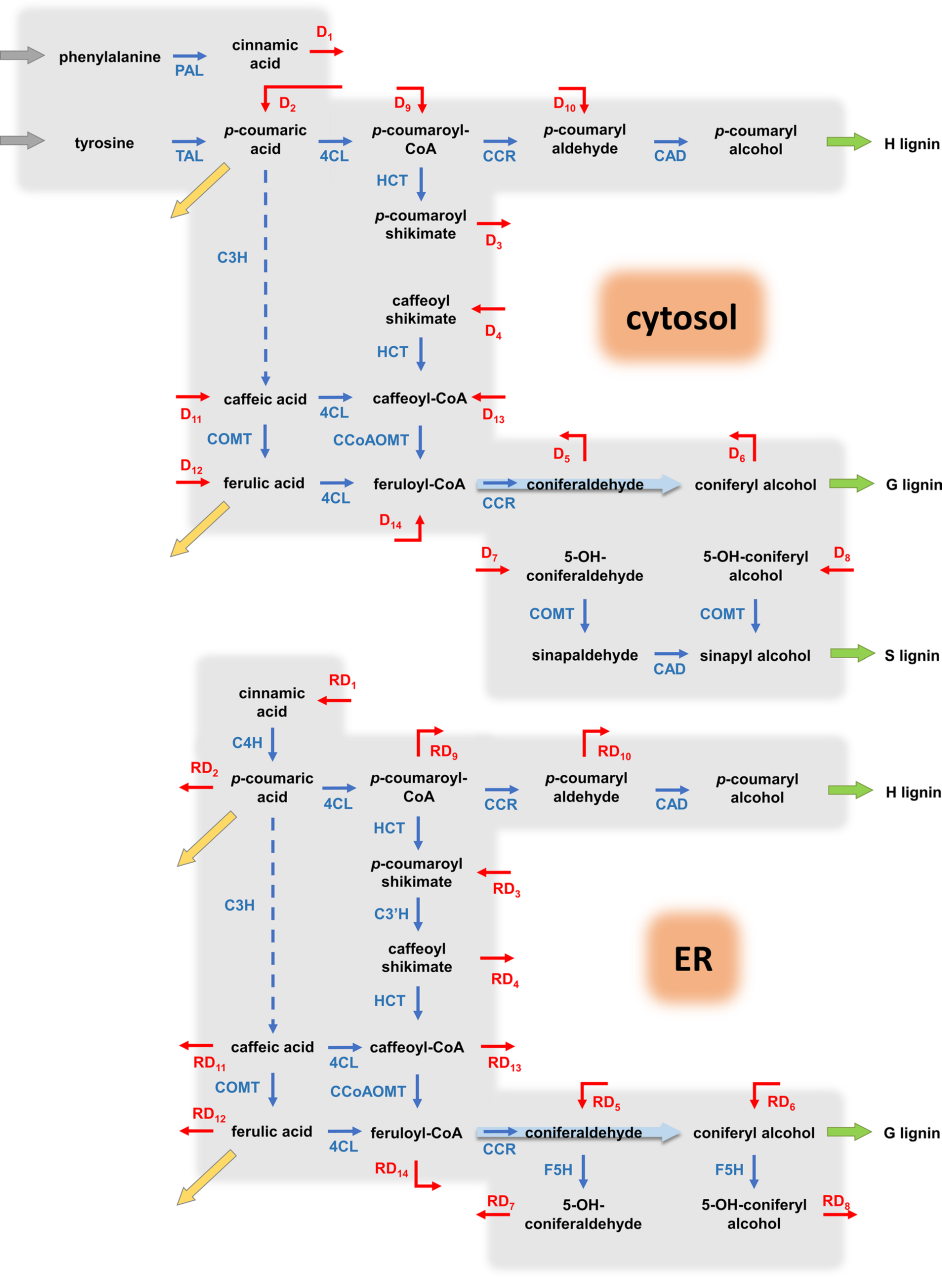
Compartmental model of the lignin pathway in *Brachypodium*. The two compartments cross-talk through diffusion fluxes (red arrows). Enzymatic reactions are marked in blue. Green arrows represent monolignol transport into the cell wall. The yellow arrows are effluxes towards wall-bound *p*-coumaric acid and ferulic acid. The coefficient *R* in expressions like $RD_{1}$ represents the ratio of volumes of the cytosol and ER compartments ($R\, = \,{r \mathord{\left/ {\vphantom {r {\left( {1\, - \,r} \right)}}} \right. \kern-0pt} {\left( {1\, - \,r} \right)}}$, where $r$ is the portion of cytosol volume and $1\, - \,r$ is the portion of ER volume with respect to the total volume). *R* is a multiplier in the rates of diffusion fluxes associated with the ER that accounts for the difference in volumes

**Fig. 3:**
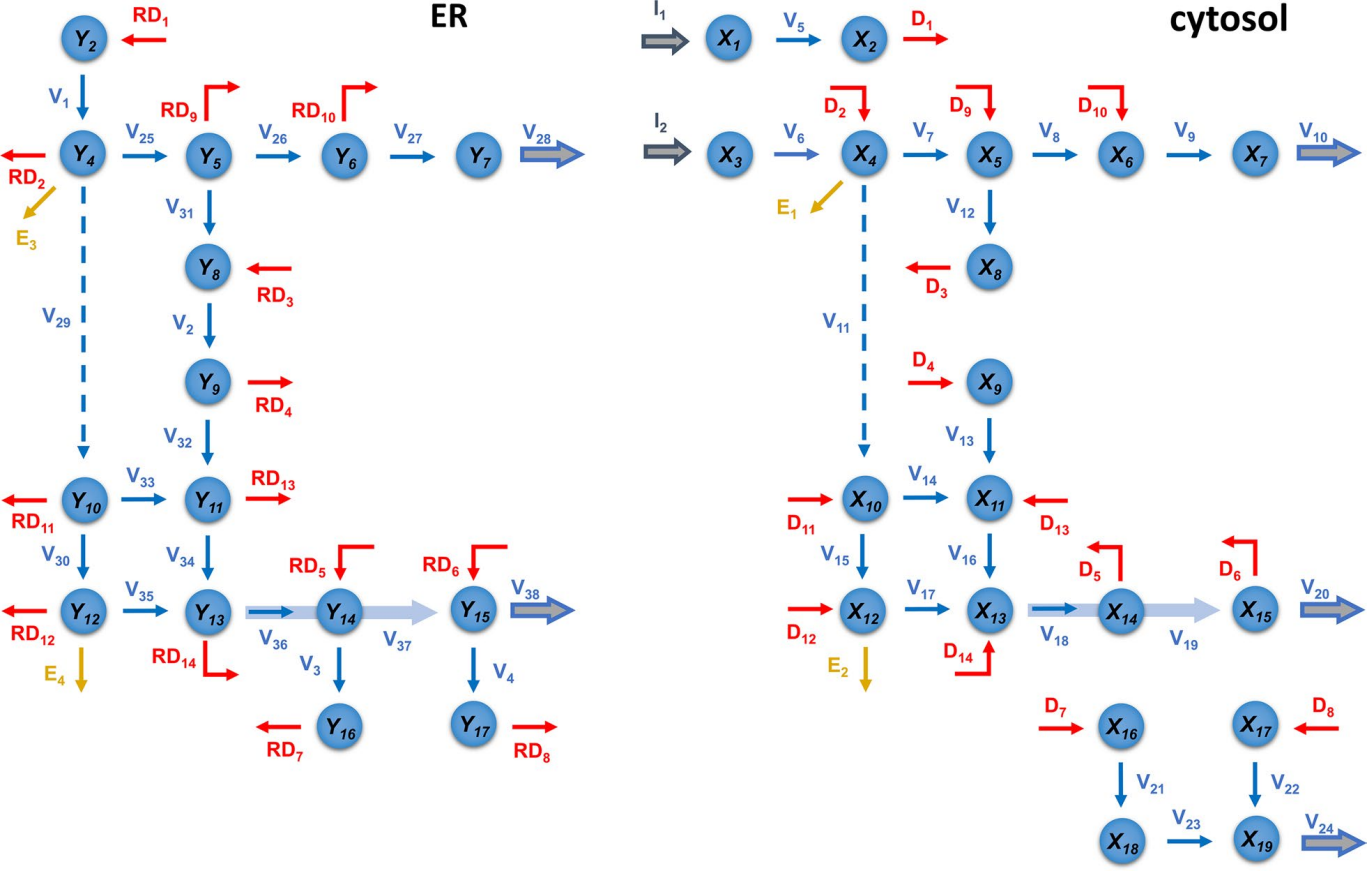
Compartmental model scheme with simplified notation. *X*_*i*_ and *Y*_*i*_ are corresponding pools of the same metabolite *i* in the cytosol and at the outer ER surface, respectively

We began our modeling efforts with the design of a one-compartment model of the pathway (Fig. 1), but soon discovered that this structure was insufficient. As a positive upshot of this initially failed analysis, the systematic exploration of the model led us to the proposal of a two-compartment model, which ultimately reproduced all data faithfully, and, in particular, captured the differential channeling of ^13^C-labeled precursors. The two “compartments” in this model are the cytosol and the outer ER surface, as discussed before. The model is shown in Fig. 2. For simplicity of notation and analysis, we rename all variables in the two compartments with subscripted *X* and *Y* (Fig. 3).

### Simulation with the static model

Computational simulations with the static model suggest that coniferaldehyde, a common precursor of both G- and S-lignin, is the critical node where the ultimate lignin composition appears to be determined. At this branch point, located on the outer ER surface, the distinct funneling of Phe and Tyr toward different lignin units is achieved through metabolic channeling, as it apparently also occurs in alfalfa and switchgrass [8, 11]. One should note that this metabolic channel does not necessarily take the form of an enzyme complex. Moreover, the wrinkled environment of the outer ER surface could provide isolated centers of metabolic activity and some physical barrier to prevent portions of the metabolites from immediate dilution by diffusion in and out of the cytosol. Whatever the natural implementation of this separation might be, it is interesting that compartmentalization appears to be a key ingredient for the preferential incorporation of ^13^C-labeling.

**Fig. 4:**
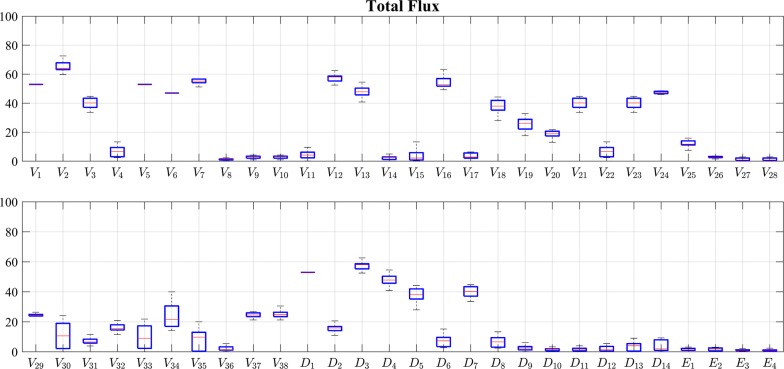
Steady-state distributions of all fluxes in the pathway, obtained from iterative simulations with the static model. The total fluxes include both labeled and unlabeled components of each flux and are independent of the labeling experiments. Each entry in these box plots shows the admissible range for each involved flux, with the center red line representing the median and the blue box containing the middle 50% of all admissible solutions. The partition coefficient *R* is factored out in this figure since it does not affect the flux profile at the steady state

**Fig. 5:**
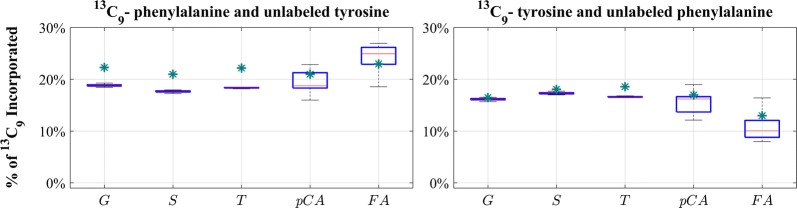
Simulated ^13^C_9_ incorporation into lignin monomers and cell wall phenolics in labeled Phe and Tyr feeding experiments. Similar to Fig. 4, this figure uses differently colored box plots to differentiate label incorporation for the two labeling experiments. The differences are quite subtle, but nevertheless collectively yield the observed incorporation preferences for Phe or Tyr substrates into different monolignols. The green asterisks represent measured ^13^C_9_ incorporation [27]

Figure 4 exhibits simulation results representing ensembles of steady-state flux distributions that were computed from the available data (see “Methods”). The box plots show the admissible range for each flux. Figure 5 details the labeled portions of the total fluxes shown in Fig. 4. The blue and red box plots compare the results for labeled Phe and Tyr supplies, respectively. It is interesting to note how different the magnitudes of the steady-state fluxes are.

### A dynamic model of lignin biosynthesis in *Brachypodium*

With the two-compartment model scheme and the computed steady-state flux distribution, we are now equipped to set up a dynamic kinetic model of the pathway, which can subsequently be used for assessing the consequences of knockdowns and other perturbations. In fact, such a model offers future opportunities for predicting responses to numerous types of alterations and, in particular, optimal genomic changes with respect to lignin content and composition (cf. [15]).

The formulation of a dynamic model is rather straightforward if one uses the modeling framework of Biochemical System Theory (BST), because every process in a BST model is represented as a product of power-law terms that reflects directly which variables are involved in this process [16–19]. Following the procedures described in *Methods*, we designed a fully dynamic model with 68 ordinary differential equations (ODE); the number 68 corresponds to twice the number of metabolites, due to formulating the system for labeled and unlabeled metabolites. The equations are presented in Additional files 1 and 2.

In stark contrast to the ease of capturing the system structure with a BST model, the determination of parameter values is a true challenge. If kinetic data on enzymes and regulators or metabolic time series data were available, one could use one of the uncounted methods that have been developed for this purpose of parameter estimation (e.g., [20–26]). Unfortunately, the information needed for these methods is not available in our case.

Instead, we used a Monte Carlo sampling method, combined with a sophisticated search algorithm, to parameterize the system such that the experimental results were satisfactorily matched (see “Methods”). The model criteria for a parameter set to be considered admissible were:

1. A good match with the observed lignin content and composition profile in control plants;
2. Consistency with the label incorporation profile in lignin monomers and in wall-bound *p*-coumaric and ferulic acid observed for labeled Phe feeding;
3. Consistency with the label incorporation profile in lignin monomers and in wall-bound *p*-coumaric and ferulic acid observed for labeled Tyr feeding;
4. A good match with the observed label incorporation profile in lignin monomers when unlabeled cinnamic acid was added to the medium.
  In labeled Phe feeding experiments and;
  In labeled Tyr feeding experiments;
5. A good match with the observed label incorporation profile in lignin monomers and wall-bound *p*-coumaric and ferulic acid when unlabeled *p*-coumaric acid was added to the medium.
  In labeled Phe feeding experiments and;
  In labeled Tyr feeding experiments.

For implementing these criteria, a match was defined as “good” if the simulation result fell within a range bounded by the mean value plus/minus 25%. We set a slightly more relaxed admissible range for wall-bound phenolics, because their effluxes have not been characterized with a sufficient degree of precision and because they are used by the plant for other purposes outside lignin production. Beyond the steady-state ensemble of flux distributions, the analysis led to an ensemble of parameter sets that rendered dynamic model simulations consistent with the experimental results.

**Fig. 6:**
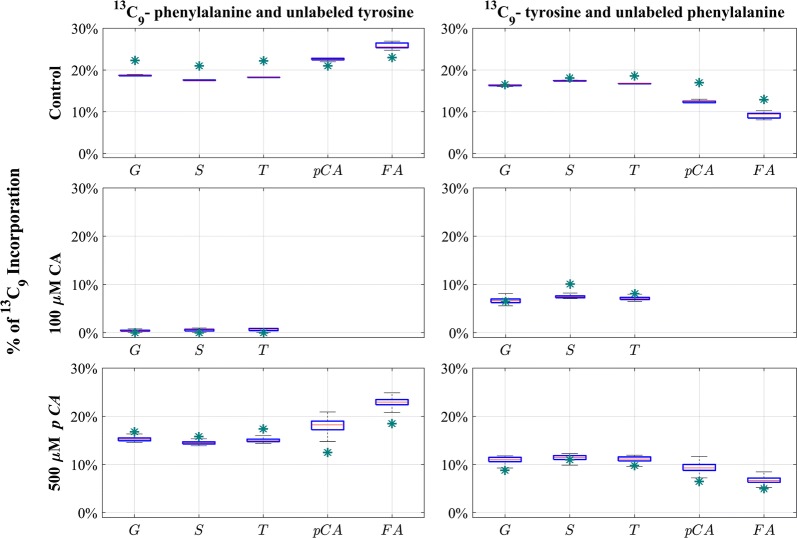
Simulated ^13^C_9_ incorporation in lignin monomers and cell wall phenolics in cinnamic acid and *p*-coumaric acid dilution experiments with labeled Phe and Tyr feeding. The parameterized dynamic model results capture label incorporation in lignin monomers and cell wall phenolics for the control plants and the plants grown on cinnamic acid and *p*-coumaric acid feeding. The green asterisks represent measured ^13^C_9_ incorporation [27]

In a separate set of experiments, Barros et al. [27] added unlabeled cinnamic acid or *p*-coumaric acid to the medium to elucidate further to what degree Phe and Tyr enter distinct reaction chains of lignin biosynthesis. Again, labeled Phe or Tyr was supplied, and the authors traced how the unlabeled cinnamic acid and *p*-coumaric acid dilute each labeling feed source. Figure 6 exhibits the parameterized dynamic model results in simulated control plants, as well as dilution experiments using labeled Phe and Tyr. As the figures indicate, the experimental data and simulations show a satisfactory match within our set criteria for acceptance.

### Validation with results from *BdPTAL* knockdown experiments

**Fig. 7:**
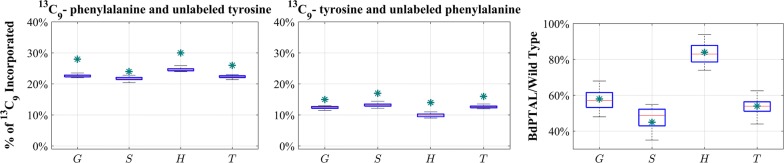
Predicted ^13^C_9_ incorporation in lignin monomers (G, S, H, and Total) and lignin composition in *BdPTAL* knockdown following labeled Phe and Tyr feeding. The left and center panels compare model predictions and measured levels of label incorporation in transgenic *BdPTAL* plants in response to labeled Phe or Tyr feeding. The right panel shows changes in lignin composition relative to wild type, and compares model predictions with measured results

The model can now be used for other predictions, e.g., regarding responses to single or double gene knockdowns, as described in [11, 15] for switchgrass. To validate the prediction accuracy of our earlier switchgrass model, we used a transgenic line whose data had not been used to construct the model, adjusted the model parameters for the measured enzyme profile in the so-far not-used perturbation experiment and compared simulated and observed lignin compositions. The model was able to capture the corresponding measured lignin composition quite well [11]. We then used the validated model to simulate single, double and global enzyme alterations and generated virtual transgenic plant libraries with altered lignin compositions. We pursued the same general strategy here, using the *BdPTAL* knockdown data (see Additional files 1 and 2), which had not been used to parameterize the model. The altered gene expression of pathway enzymes and the lignin composition were available. We used the expression changes as changes in enzyme activities and simulated the transgenic plant. Figure 7 shows the simulation results. As the figure demonstrates, simulations and experimental data match quite well. We suspect that the slight underestimation in label incorporation might be due to the lack of data on Phe and Tyr label levels in these experiments, which required us to use the measured Phe and Tyr label levels from the dilution experiments, which may be slightly different. Nonetheless, the data are matched at least semi-quantitatively.

## Methods

### Static model

The construction of the compartmental model and methods for estimating the steady-state flux distribution for *Brachypodium* have been discussed elsewhere [13]. To estimate the cinnamic acid (CA) and *p*-coumaric acid (*p*CA) input that is actually taken up by the plants from the medium and enters the lignin pathway, we used the steady-state model and recorded all input values of CA and *p*CA that led to lignin and wall-bound phenolic pro**file**s matching the experimental data. These data came from labeling experiments in wild-type lines and include labeled and unlabeled lignin monomer profiles, as well as the label incorporation level in wall-bound *p*-coumaric acid and ferulic acid [27]. We used the estimated flux distribution, as well as CA and *p*CA input values to parameterize the dynamic model.

### Construction of the dynamic model

#### Model format

We designed our dynamic model as a system of ordinary differential equations (ODE), taking the metabolites as the states of the system. The enzymatic fluxes are modeled under the tenets of biological systems theory (BST) in generalized mass action (GMA) format [16–19]. These base methods are explained in detail in our previous work on switchgrass [11]. The compartmental model proposed here additionally includes diffusion fluxes, which transfer mass between the compartments (Fig. 2). These net diffusion fluxes are modeled in the format

1 $$D_{j} \, = \,d_{j} \left( {X_{i} \, - \,Y_{i} } \right),$$

where $X_{i}$ and $Y_{i}$ are corresponding pools of the same metabolite in the cytosol and outer ER surface compartments, respectively, and $d_{j}$ is the rate constant of diffusion.

Due to the lack of specific literature on metabolite profiles, and similar to our approach for switchgrass, the concentrations are normalized with respect to the wild-type steady-state value, and the base value is set to 100:

2 $$\begin{aligned} X_{i} \, = \,\frac{100}{{Z_{{i , {\text{SS}}}} }} \cdot Z_{{i , {\text{cyt}}}} , \hfill \\ Y_{i} \, = \,\frac{100}{{Z_{{i , {\text{SS}}}} }} \cdot Z_{{i , {\text{ER}}}} , \hfill \\ \end{aligned}$$

where $Z_{{i , {\text{SS}}}}$ is the nominal total concentration of metabolite $i$ at steady state, and $Z_{{i , {\text{cyt}}}}$ and $Z_{{i,{\text{ER}}}}$ are local concentrations of metabolite $i$ in the respective compartments. Expressed differently, $X_{\text{i}}$ and $Y_{\text{i}}$ are the local, normalized concentrations of metabolite $i$ in each compartment. At the steady state, the total concentration of each metabolite is equal to 100,

3 $$r \cdot X_{i} \, + \,\left( {1\, - \,r} \right) \cdot Y_{i} \, = \,100 ,$$

where $r$ is the portion of the cytosol compartment volume and $1\, - \,r$ is the portion of the ER compartment volume with respect to total volume. Hence, ${r \mathord{\left/ {\vphantom {r {\left( {1\, - \,r} \right)}}} \right. \kern-0pt} {\left( {1\, - \,r} \right)}}$ is the ratio of volumes earlier introduced as $R$. We set $r$ equal to 0.9, which corresponds to the cytosol compartment accounting for 90% and the ER outer surface compartment to 10% of the cell volume within which the lignin pathway is active.

#### Equations for labeled and unlabeled substrates

We extended the dynamic base model to a system accounting for labeled and unlabeled metabolites, which doubled the number of ODEs. Each enzymatic flux $V_{j}$ is calculated using the total metabolite pool whether labeled or unlabeled; in other words, the enzymes are assumed to be blind to labels:

4 $$V_{j} \, = \,a_{j} \prod\limits_{i = 1}^{n} {\left( {X_{i,L} \, + \,X_{i,UL} } \right)^{{g_{i,j} }} } \prod\limits_{i = n + 1}^{n + m} {X_{i}^{{h_{i,j} }} } .$$

where $a_{j}$ is the rate constant, $X_{i,L}$ and $X_{i,UL}$ ($1 < i < n$) are labeled and unlabeled metabolites, respectively, and $g_{i,j}$’s are kinetic orders. For $n\, + \,1\, < \,i\, < \,n\, + \,m$, each variable $X_{i}$ represents the amount of an enzyme involved in the reaction. It is customary to assign enzyme kinetic orders, $h_{i,j}$, equal to 1 if the enzyme is involved in the reaction and 0 if it is not. $X_{i}$ is replaced by $Y_{i}$ if the reaction is taking place in the ER compartment. The labeled and unlabeled flux portions are calculated based on how rich in labels the substrate pool of the flux is (Eq. 5).

5 $$\begin{aligned} V_{j,L} \, = \,\frac{{X_{s,L} }}{{X_{s} }} \cdot V_{j} , \hfill \\ V_{j,UL} \, = \,\frac{{X_{s,UL} }}{{X_{s} }} \cdot V_{j} , \hfill \\ \end{aligned}$$

where $X_{s}$ is the substrate of the reaction $V_{j}$. To compute the rate of change in each metabolite, the labeled fluxes are used to establish the ODEs for labeled metabolites, and the unlabeled fluxes are used to formulate the differential equations of the unlabeled metabolites. As an example, the equations for labeled and unlabeled *p*-coumaroyl-CoA in the ER compartment are written as

6 $$\begin{aligned} & \frac{{dY_{5,L} }}{dt}\, = \,\frac{{Y_{4,L} }}{{Y_{4} }} \cdot V_{25} \, - \,\frac{{Y_{5,L} }}{{Y_{5} }} \cdot V_{26} \, - \,\frac{{Y_{5,L} }}{{Y_{5} }} \cdot V_{31} \, - \,\frac{r}{1\, - \,r}d_{9} \left( {Y_{5,L} \, - \,X_{5,L} } \right), \\ & \frac{{dY_{5,UL} }}{dt}\, = \,\frac{{Y_{4,UL} }}{{Y_{4} }} \cdot V_{25} \, - \,\frac{{Y_{5,UL} }}{{Y_{5} }} \cdot V_{26} \, - \,\frac{{Y_{5,UL} }}{{Y_{5} }} \cdot V_{31} \, - \,\frac{r}{1 - r}d_{9} \left( {Y_{5,UL} \, - \,X_{5,UL} } \right). \\ \end{aligned}$$

#### Unknowns of the system and parameterization

The values of the kinetic orders, $g_{i,j}$, rate constants,$a_{j}$, $d_{j}$, and steady-state values of the parallel metabolite pools, $X_{{i,{\text{SS}}}}$ and $Y_{{i,{\text{SS}}}}$, are unknown and need to be estimated. To estimate the unknowns, we used Monte Carlo sampling, generating a random set sampled from the space ℝ^*p*^, and within the biologically reasonable ranges, where *p* is the number of unknowns.

Most of the metabolite steady-state values can be algebraically computed as follows. We can rewrite Eq. 1 for the labeled portion of diffusion flux as

7 $$D_{j,L} \, = \,d_{j} \left( {L_{{X_{i} }} \cdot X_{i} \, - \,L_{{Y_{i} }} \cdot Y_{i} } \right),$$

where $L_{{X_{i} }}$ and $L_{{Y_{i} }}$ are label incorporation levels in $X_{i}$ and $Y_{i}$ pools, which are known from the labeled flux distributions [13]. From Eqs. 1, 3 and 7 together, the three unknowns $d_{j}$,$X_{i}$ and $Y_{i}$ can be algebraically computed for each diffusion flux. Due to the stoichiometry of the pathway, four of the metabolites, namely cinnamic acid ($X_{2}$,$Y_{2}$), caffeoyl shikimate ($X_{9}$, $Y_{9}$), 5-OH-coniferaldehyde ($X_{16}$, $Y_{16}$), and 5-OH-coniferyl alcohol ($X_{17}$, $Y_{17}$), have the same level of label incorporation in both of their cytosol and ER compartments. That leads to a degenerate case where Eqs. 1 and 7 are linearly dependent ($L_{{X_{i} }} \, = \,L_{{Y_{i} }}$), and steady-state values cannot be computed algebraically. Hence, the steady-state values of these metabolite pools, $\left( {X_{{2,{\text{SS}}}} ,Y_{{2,{\text{SS}}}} } \right)$, $\left( {X_{{9,{\text{SS}}}} ,Y_{{9,{\text{SS}}}} } \right)$, $\left( {X_{{16,{\text{SS}}}} ,Y_{{16,{\text{SS}}}} } \right)$, $\left( {X_{{17,{\text{SS}}}} ,Y_{{17,{\text{SS}}}} } \right)$, need to be estimated. Since the total concentrations of each pair of metabolites ($r \cdot X_{i} \, + \,\left( {1\, - \,r} \right) \cdot Y_{i}$) are equal to 100 at the steady state, it suffices to estimate either $X_{{i,{\text{SS}}}}$ or $Y_{{i,{\text{SS}}}}$ for parallel metabolite pools, and use Eq. 3 to compute the other. Given the steady-state flux distribution, the diffusion rate constants, $d_{j}$, are then computed using Eq. 1. To sample the steady-state metabolite values, we considered the direction of diffusion between $X_{{i,{\text{SS}}}}$ and $Y_{{i,{\text{SS}}}}$. As Eq. 3 shows, the weighted average of $X_{{i,{\text{SS}}}}$ and $Y_{{i,{\text{SS}}}}$ is equal to 100 at the steady state, which implies that one of the pools is greater than or equal to 100, and the other smaller than or equal to 100. The direction of diffusion determines the pool smaller than 100 since the diffusion flux pours into that pool at the steady state. This way, we obtain an interval for sampling bounded between zero and 100. Therefore, we sampled the array

8 $$\left[ {Y_{{2,{\text{SS}}}} ,X_{{9,{\text{SS}}}} ,X_{{16,{\text{SS}}}} ,X_{{17,{\text{SS}}}} } \right]$$

within [0,100] and computed the corresponding parallel pools, $\left[ {X_{{2,{\text{SS}}}} ,Y_{{9,{\text{SS}}}} ,Y_{{16,{\text{SS}}}} ,Y_{{17,{\text{SS}}}} } \right]$, using Eq. 3. In fact, the pools of metabolites in Eq. 8 are the pools into which the diffusion fluxes pour at the steady state (Fig. 2).

Kinetic orders *g*_*i,j*_ were sampled from the interval [0,1] if metabolite *i* was a substrate or activator of flux *j* (except for effluxes *E*_1_*, E*_2_*, E*_3_ and *E*_4_ for which we used the interval [0, 4]), and [− 4,0] if they acted as inhibitors of flux *j* [28]. Using the steady-state flux and metabolite values and the randomly generated kinetic orders, *g*_*i,j*_, the rate constants, *a*_*j*_, of the enzymatic reactions are then computed via Eq. 4.

Due to the high number of unknown parameters, dilution experiments were parameterized one at a time. The thus obtained working parameters from one experiment were used to regenerate new parameters in the reduced parameter space, employing random Monte Carlo sampling. Therefore, hundreds of thousands of parameter combinations were simulated to satisfy all model criteria.

Specifically, we used a variation of an explore-and-exploit algorithm [29–31], starting with an initial, randomly generated parameter set. This algorithm is designed to target those areas of the parameter space that are in the vicinity of an admissible solution once such a solution is found; it exploits these neighborhoods rather than randomly exploring the parameter space at large. In other words, the algorithm benefits from the search history and performs a more effective optimization within promising, localized domains of the parameter space. Details of our implementation of the algorithm are discussed in Additional files 1 and 2.

For the dynamic model, the criteria of admissibility were the same as the criteria in the static model. Thus, parameter values were deemed admissible if they met the model criteria presented in “Results” section. Namely, they had to yield a good representation of the observed lignin monomer composition and total lignin amount, and match the label incorporation profiles in lignin monomers, as well as in wall-bound *p*-coumaric acid and ferulic acid to a satisfactory degree.

### Dynamic model validation

#### Enzyme activity estimation from changes in gene expression

Ideally, it would be possible to determine the concentrations and activities of all enzymes involved in the pathway. However, in situ measurements of protein concentrations of the magnitudes available in lignin biosynthesis are extremely difficult to obtain, and as of yet no reliable information is available. It, therefore, appears that reliable and rather precise transcripts of genes coding for the enzymes of interest are our best option.

**Fig. 8:**
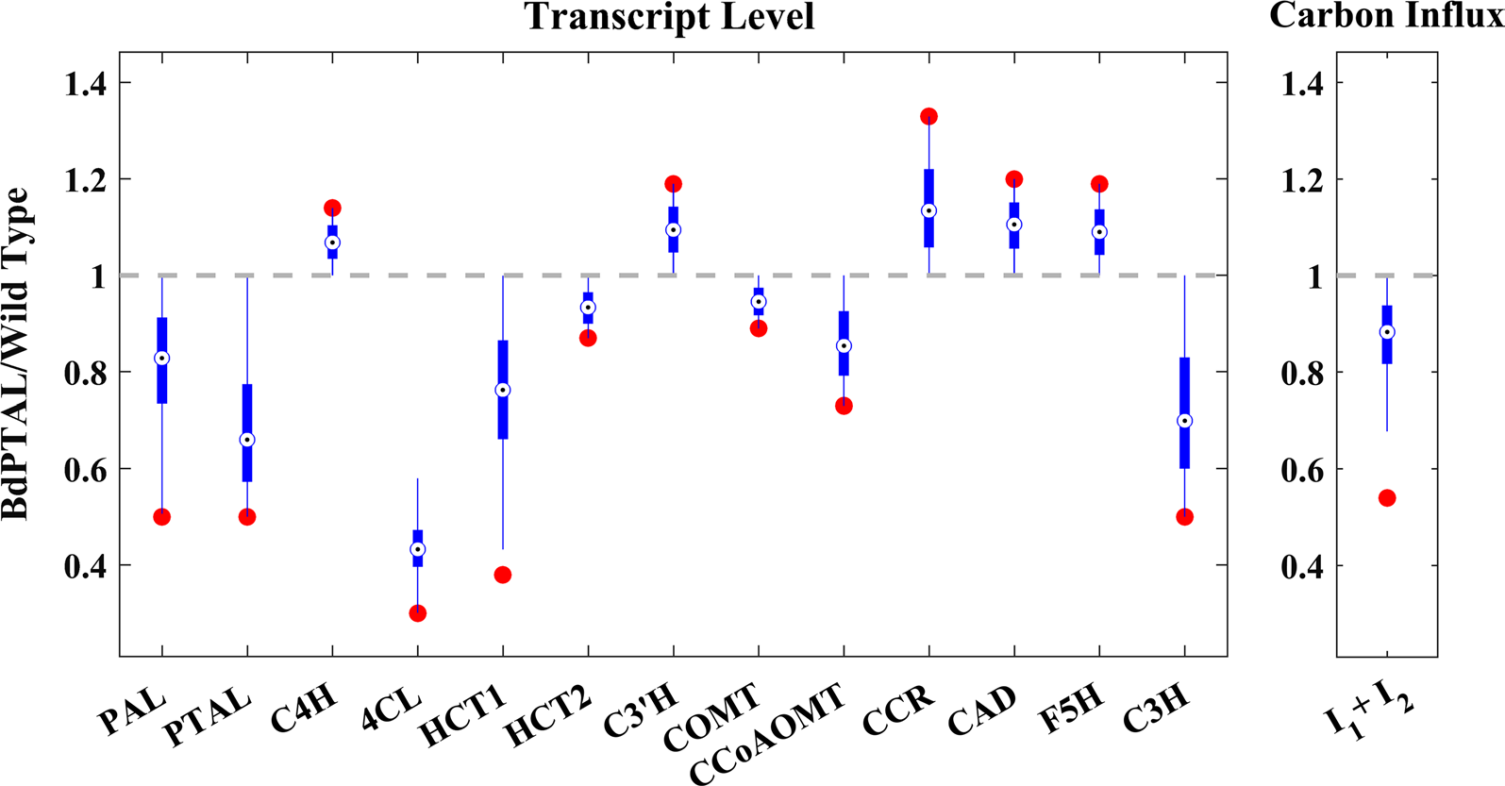
*BdPTAL* transcript profile and simulated admissible enzyme activity profile. In the left panel red circles represent measured transcript levels in *BdPTAL* relative to wild type. Blue box plots are the simulated admissible ranges of enzyme activity levels in *BdPTAL* relative to wild type. The gray dashed line marks no change with respect to wild type. Note that the plot in some sense compares apples (observed transcript changes) and oranges (enzyme activity changes that are admissible according to the model analysis). The right panel depicts the change in carbon influx into the system relative to wild type

It is known that changes in gene expression are not necessarily translated one-to-one measure into the corresponding enzyme activity, and it is in fact likely that a *p*% change in transcript results in a change in enzyme activity that is much lower than *p*%. To account for this uncertainty, we used the measured transcript profile of *BdPTAL* transgenics as an upper bound for the corresponding enzyme activity levels. Thus, we randomly generated 20,000 altered enzyme activity profiles bounded between the wild type and *BdPTAL* transcript level, simulated all these profiles with the dynamic model, and retained those close enough to the *BdPTAL* observations of lignin amounts and labeling levels. Figure 8 shows the altered enzyme activity profiles that yielded admissible lignin composition profiles. In addition to the enzyme transcripts, the right panel depicts the changes in carbon influx relative to wild type in the admissible solutions. Although the enzyme transcripts of the upstream pathway are not measured in this study, we included this extra measure in our model to examine the necessity of an orchestrated decrease in carbon influx into the pathway by the plant. The results indicate that the median of the change is at 90%, which leads to the conclusion that the influx is not strongly redirected before entering the pathway.

## Discussion

Lignin is a fascinating organic compound that is essential to the life of terrestrial plants. Its molecular composition and structural toughness are critical ingredients for the roles of lignin in plants and they are also increasingly valued by industry, which has begun to use lignin as a starting substrate for a variety of products [32].

Both targeted reductions and increases in lignin, as well as any changes in its molecular composition, require a detailed understanding of how lignin is synthesized in vivo. This understanding is difficult to gain from wet experiments alone, as the biosynthetic lignin pathway is complex and reuses the same enzymes several times for different substrates. This multiple use makes intuitive predictions regarding introduced alterations in these enzymes difficult and unreliable. Not surprisingly, the pathway also contains regulation, as well as functional channels, which permit some control over the flux into specific monolignols, and these nonlinear features confound explanation and prediction capabilities. In addition to these features, we have suggested here and elsewhere [13] that the pathway in *Brachypodium* appears to be functionally separated into two locations, namely the cytosol and the outer surface of the ER. Otherwise, the *Brachypodium* pathway seems similar—although not entirely identical—to the corresponding pathways in alfalfa and switchgrass. The assumption of different locations for enzymatic activity actually seems very reasonable, because experimental observations report that three of the pathway enzymes, namely C4H, C3′H and F5H, are bound to the outer surface of ER, whereas other enzymes are commonly assumed to be free in the cytosol. This has been reported in *Brachypodium* [27], tobacco [33], and *Arabidopsis* [34]. Whether a similar compartmentalization exists in most or all terrestrial plant species is not known and will require targeted experimentation.

In terms of different species and their own peculiarities with respect to the lignin pathway, experimental work suggests that there are indeed distinctions with respect to the presence or absence of some metabolites and reactions. For instance, alfalfa converts caffeoyl-CoA into caffeoyl aldehyde by means of CCR, and COMT catalyzes the subsequent reaction converting caffeoyl aldehyde into coniferaldehyde [35]. These two reactions are apparently absent in switchgrass, black cottonwood, and *Brachypodium*. Also, in switchgrass and *Medicago truncatula*, caffeoyl shikimate esterase (CSE) converts caffeoyl shikimate into caffeic acid [36], while this reaction seems not to be functional in either *Brachypodium* or black cottonwood. Specifically for *Brachypodium*, it was, furthermore, shown that phenylalanine (Phe) is not the only substrate for the lignin pathway, but that this organism can also use tyrosine (Tyr) in almost equal amounts. It is unknown whether this secondary pathway is a matter of redundancy, whether there are specific internal or environmental reasons for this organism needing this alternative, or whether the organism uses these pathways to control its S/G ratio.

Over the past decade, we have developed models of lignin biosynthesis in different plant species [8–11, 13]. These models not only contain similar features, such as a common reaction “skeleton” and certain metabolic channels, but also exhibit differences. For instance, the model for switchgrass requires feedback inhibition by reaction products, whereas the model for *Brachypodium* is only consistent with the available data if it is distributed over two compartments. While the similarities are encouraging toward a streamlined understanding of lignin biosynthesis, the differences should not be overinterpreted. It is quite possible that the pathways in alfalfa and switchgrass might be compartmentalized as well, and that products inhibit reactions in *Brachypodium*, but our modeling efforts, adhering to the simplicity of Ockham’s razor, do not require these features, possibly only because the same types of data are not available for the various species. Thus, much further exploration and analysis will be needed to determine whether all models designed so far are special cases of one common model that simultaneously contains all these features or whether evolution has led to distinct implementations of a basic model structure with species-specific variations that respond to different environmental demands.

While it will be interesting to explore similarities and true differences among a variety of species further, it is becoming clear that, even in the face of scarce data and substantial information gaps, dynamic models are gaining in relevance and importance. They not only integrate different datasets and other auxiliary information, but they are in the process of becoming obligatory tools for making reliable predictions regarding natural and introduced alterations in the metabolic pathway systems that generate lignin in different organisms.

## Additional files

**Additional file 1.** Additional materials.

**Additional file 2.** Additional table.
