# Supplementary material for: A dynamic model of lignin biosynthesis in Brachypodium distachyon
Source: Biotechnol Biofuels. 2018 Sep 19;11:253. doi: 10.1186/s13068-018-1241-6 (PMC6145374; doi:10.1186/s13068-018-1241-6)
Supplement: Supplementary file 1 — Additional file 1. Additional materials. [file 13068_2018_1241_MOESM1_ESM.docx]

**Additional file 1**

**A Dynamic Model of Lignin Biosynthesis in *Brachypodium distachyon***

**Mojdeh Faraji, Luis L. Fonseca, Luis Escamilla-Treviño, Jaime Barros-Rios, Nancy L. Engle, Zamin K. Yang, Timothy J. Tschaplinski, Richard A. Dixon, Eberhard O. Voit**

**Explore-and-exploit algorithm**

This parameter estimation algorithm has two modes, called *explore* and *exploit*. A simulation begins with a parameter set, containing *N* randomly generated parameters. Since the parameters are generated randomly, each entry of the parameter set is at a random location in parameter space without a specific order. Therefore, simulations initially explore the parameter space randomly as well. Exploration continues until a solution is found. Namely, when parameter *i* satisfies previously defined model criteria. Then the algorithm starts exploiting the space around this solution, rather than continuing random exploration of the rest of the parameter set. In order to exploit the vicinity of the solution, the parameters from the initial set that have not been simulated yet (parameters *i* + 1 : *N*) are sorted by distance with respect to the first solution found (parameter *i*). Since other clusters of solutions might exist, only a certain radius from this solution is exploited. We call this distance exploit radius, and define it as the distance which contains the closest *m* parameters to the first solution within the unexplored set (parameters *i* + 1 : *N*). We choose m = 0.1*N*. When more solutions are found, the center of gravity of solutions (CG) is used to set the exploit radius.

To improve the efficiency of the algorithm, we reorganize the exploit set as follows. In a nonlinear system, it is not surprising to have a nonconvex solution set. Due to this common phenomenon, we re-sort parameters in the exploit set (parameters *i* + 1 : 0.1*N*) by distance with respect to the last solution found, *i.e.*, parameter *i*. This way, the priority of the search is placed on the parameters closest to the last solution found rather than the ones closet to the CG of solutions. Parameters outside the exploit set (parameters 0.1*N* + 1 : N) remain unsorted and retain a random order (Figure S1).

The simulation resumes with the new parameter set and within the exploit set. If a new solution is found, the steps above are repeated. If no solution is found within the exploit set, the algorithm automatically starts exploring the parameter space randomly since parameters outside exploit set (parameters 0.1*N* + 1 : N) are in random order. A typical stop criterion is met when half of the initial parameter set (0.5*N*) is simulated and the algorithm is back on explore mode. If no solution is found at all, algorithm explores the entire parameter set. The flowchart of the algorithm is shown in Figure S2.


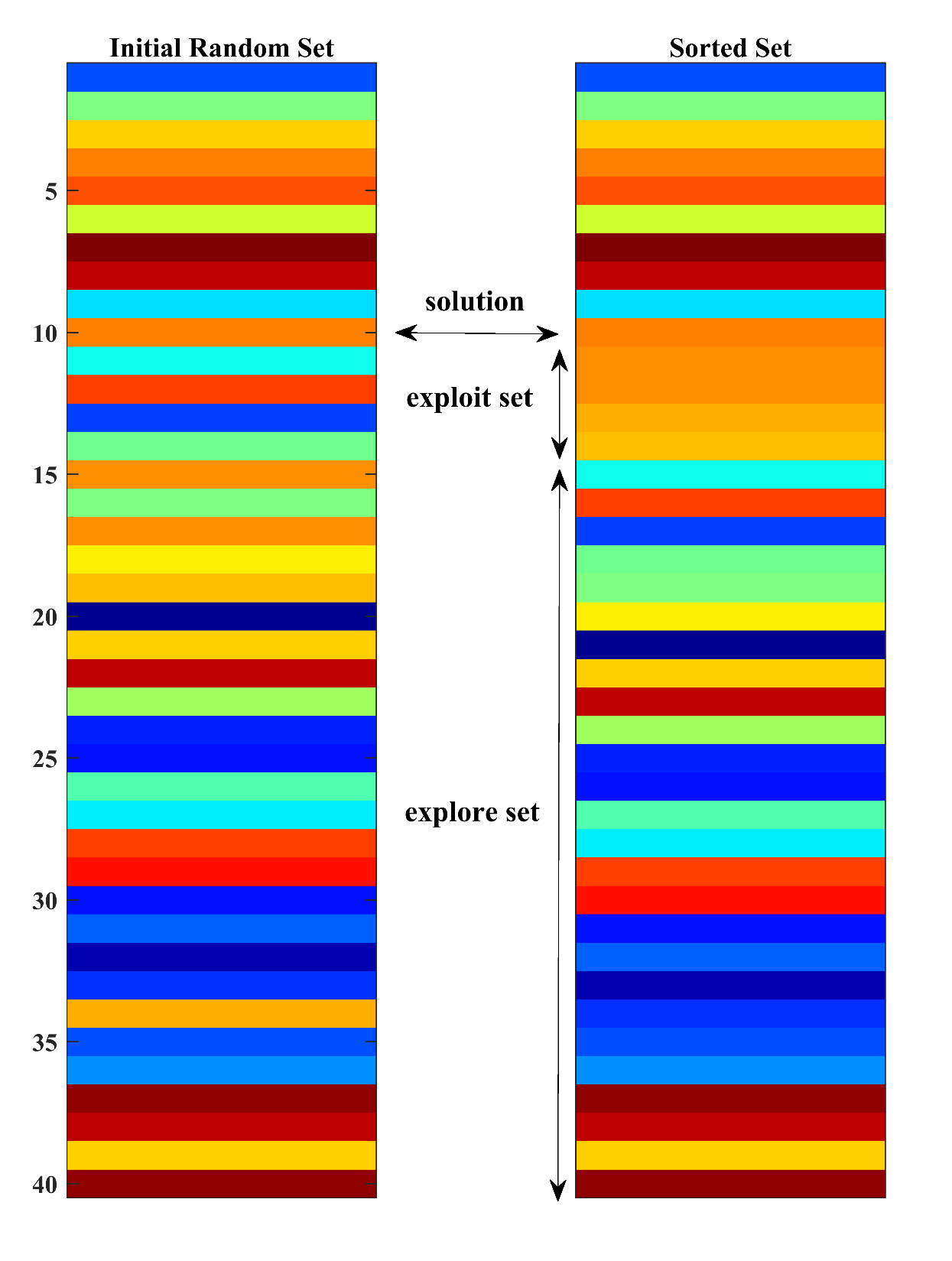


**Figure S1. Rearrangement of parameters in the explore-and-exploit algorithm.** In this illustration, parameters are color coded based on the distance from the origin. The right panel demonstrates rearrangement of the parameter set when a solution is found (i=10). The new order is such that the exploit set with the closest 0.1N parameters to the last solution found come first and then is the explore set which includes the random parameters outside the exploit set.


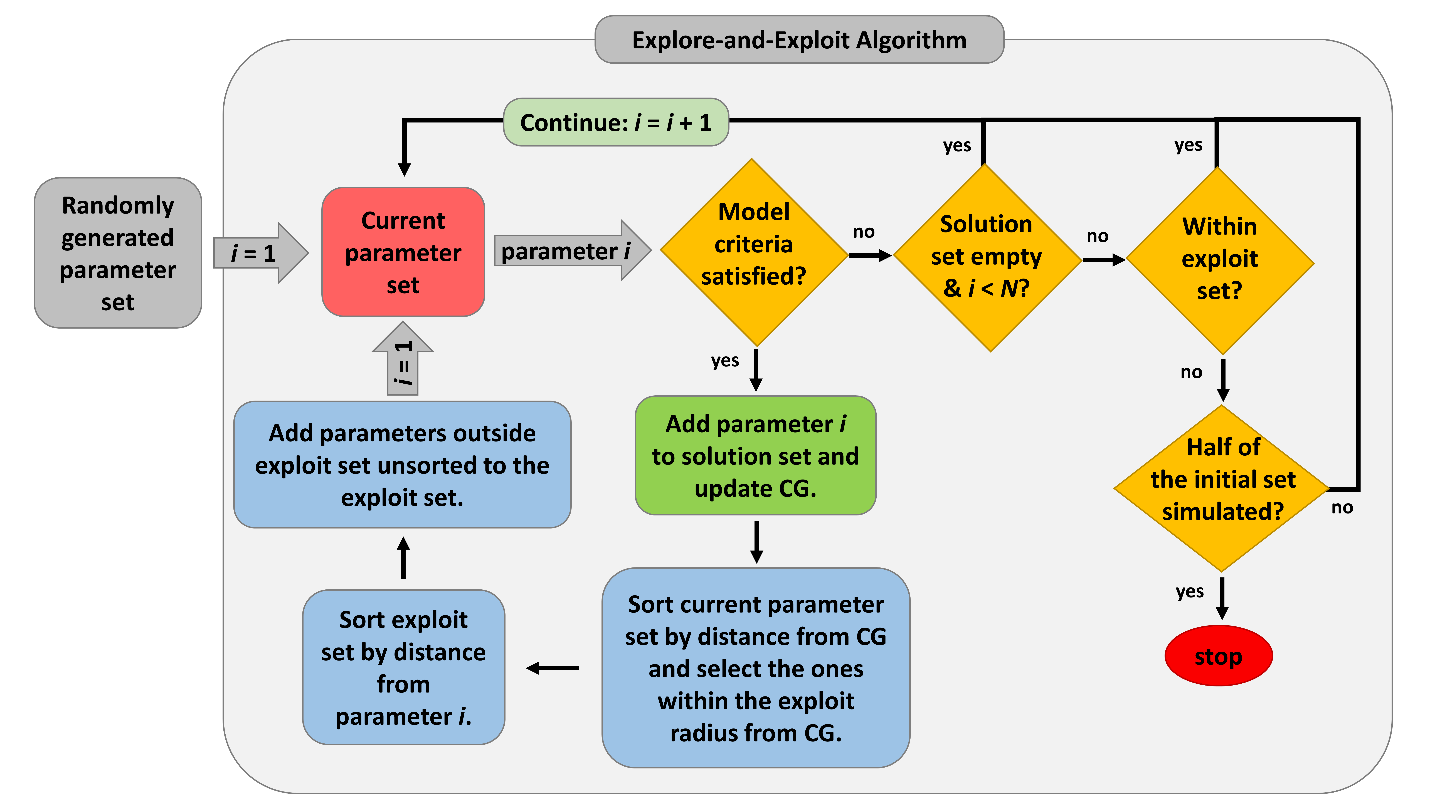


**Figure S2. Flowchart of the explore-and-exploit algorithm.**

**Equations**

The differential equations of the system corresponding to Figure 3 are formulate as follows:

*Cytosol compartment:*

$\begin{matrix} \begin{matrix} \frac{dX_{1}}{dt}=I_{1}-V_{5}-W_{X_{1}}, \\ \frac{dX_{2}}{dt}=V_{5}-D_{1}, \\ \frac{dX_{3}}{dt}=I_{2}-V_{6}-W_{X_{3}}, \\ \frac{dX_{4}}{dt}=V_{6}+D_{2}-V_{7}-V_{11}-E_{1}, \\ \frac{dX_{5}}{dt}=V_{7}+D_{9}-V_{8}-V_{12}, \\ \frac{dX_{6}}{dt}=V_{8}+D_{10}-V_{9}, \\ \frac{dX_{7}}{dt}=V_{9}-V_{10}, \\ \frac{dX_{8}}{dt}=V_{12}-D_{3}, \\ \frac{dX_{9}}{dt}=D_{4}-V_{13}, \\ \frac{dX_{10}}{dt}=V_{11}+D_{11}-V_{14}-V_{15}, \end{matrix} & & \begin{matrix} \frac{dX_{11}}{dt}=V_{13}+V_{14}+D_{13}-V_{16}, \\ \frac{dX_{12}}{dt}=V_{15}+D_{12}-V_{17}-E_{2}, \\ \frac{dX_{13}}{dt}=V_{16}+V_{17}+D_{14}-V_{18}-V_{19}, \\ \frac{dX_{14}}{dt}=V_{18}-D_{5}, \\ \frac{dX_{15}}{dt}=V_{19}-V_{20}-D_{6}, \\ \frac{dX_{16}}{dt}=D_{7}-V_{21}, \\ \frac{dX_{17}}{dt}=D_{8}-V_{22}, \\ \frac{dX_{18}}{dt}=V_{21}-V_{23}, \\ \frac{dX_{19}}{dt}=V_{22}+V_{23}-V_{24}, \\ \end{matrix} \end{matrix}$ (1)

where $R=\frac{r}{1-r}$ is the ratio of compartment volumes, and efflux $W_{X_{i}}$ is defined as

$W_{X_{i}}=\left\{\begin{matrix} 0 & , & X_{i}\leq X_{i,SS} \\ & & \\ 0.8\left(X_{i}-X_{i,SS}\right) & , & X_{i}>X_{i,SS}. \end{matrix}\right.$ (2)

*ER compartment:*

$\begin{matrix} \begin{matrix} Y_{1}=0, \\ \frac{dY_{2}}{dt}=RD_{1}-V_{1}, \\ Y_{3}=0, \\ \frac{dY_{4}}{dt}=V_{1}-V_{25}-V_{29}-RD_{2}-E_{3}, \\ \frac{dY_{5}}{dt}=V_{25}-V_{26}-V_{31}-RD_{9}, \\ \frac{dY_{6}}{dt}=V_{26}-V_{27}-RD_{10}, \\ \frac{dY_{7}}{dt}=V_{27}-V_{28}, \\ \frac{dY_{8}}{dt}=V_{31}+RD_{3}-V_{2}, \\ \frac{dY_{9}}{dt}=V_{2}-V_{32}-RD_{4}, \\ \frac{dY_{10}}{dt}=V_{29}-V_{30}-V_{33}-RD_{11}, \end{matrix} & & \begin{matrix} \frac{dY_{11}}{dt}=V_{32}+V_{33}-V_{34}-RD_{13}, \\ \frac{dY_{12}}{dt}=V_{30}-V_{35}-RD_{12}-E_{4}, \\ \frac{dY_{13}}{dt}=V_{34}+V_{35}-V_{36}-V_{37}-RD_{14}, \\ \frac{dY_{14}}{dt}=V_{36}+RD_{5}-V_{3}, \\ \frac{dY_{15}}{dt}=V_{37}+RD_{6}-V_{4}-V_{38}, \\ \frac{dY_{16}}{dt}=V_{3}-RD_{7}, \\ \frac{dY_{17}}{dt}=V_{4}-RD_{8}, \\ Y_{18}=0, \\ Y_{19}=0, \end{matrix} \end{matrix}$ (3)

The fluxes are

$\begin{matrix} \begin{matrix} V_{1}=a_{1}Y_{2}^{g_{1,1}}Z_{1}, \\ V_{2}=a_{2}Y_{8}^{g_{3,2}}Z_{2}, \\ V_{3}=a_{3}Y_{14}^{g_{5,3}}Z_{3}, \\ V_{4}=a_{4}Y_{15}^{g_{6,4}}Z_{4}, \\ V_{5}=a_{5}X_{1}^{g_{9,5}}X_{2}^{g_{10,5}}Z_{5}, \\ V_{6}=a_{6}X_{3}^{g_{11,6}}X_{2}^{g_{10,6}}X_{4}^{g_{12,6}}Z_{6}, \\ V_{7}=a_{7}X_{4}^{g_{12,7}}Z_{7}, \\ V_{8}=a_{8}X_{5}^{g_{13,8}}Z_{8}, \\ V_{9}=a_{9}X_{6}^{g_{14,9}}Z_{9}, \\ V_{10}=a_{10}X_{7}^{g_{15,10}}Z_{10}, \\ V_{11}=a_{11}X_{4}^{g_{12,11}}Z_{11}, \\ V_{12}=a_{12}X_{5}^{g_{13,12}}Z_{12}, \\ V_{13}=a_{13}X_{9}^{g_{17,13}}Z_{13}, \end{matrix} & & \begin{matrix} V_{14}=a_{14}X_{10}^{g_{18,14}}Z_{14}, \\ V_{15}=a_{15}X_{10}^{g_{18,15}}Z_{15}, \\ V_{16}=a_{16}X_{11}^{g_{19,16}}Z_{16}, \\ V_{17}=a_{17}X_{12}^{g_{20,17}}Z_{17}, \\ V_{18}=a_{18}X_{13}^{g_{21,18}}Z_{18}, \\ V_{19}=a_{19}X_{13}^{g_{22,19}}Z_{19}, \\ V_{20}=a_{20}X_{15}^{g_{23,20}}Z_{20}, \\ V_{21}=a_{21}X_{16}^{g_{24,21}}Z_{21}, \\ V_{22}=a_{22}X_{17}^{g_{25,22}}Z_{22}, \\ V_{23}=a_{23}X_{18}^{g_{26,23}}Z_{23}, \\ V_{24}=a_{24}X_{19}^{g_{27,24}}Z_{24}, \\ V_{25}=a_{25}Y_{4}^{g_{2,25}}Z_{25}, \\ V_{26}=a_{26}Y_{5}^{g_{28,26}}Z_{26}, \end{matrix} & & \begin{matrix} V_{27}=a_{27}Y_{6}^{g_{29,27}}Z_{27}, \\ V_{28}=a_{28}Y_{7}^{g_{30,28}}Z_{28}, \\ V_{29}=a_{29}Y_{4}^{g_{2,29}}Z_{29}, \\ V_{30}=a_{30}Y_{10}^{g_{31,30}}Z_{30}, \\ V_{31}=a_{31}Y_{5}^{g_{28,31}}Z_{31}, \\ V_{32}=a_{32}Y_{9}^{g_{4,32}}Z_{32}, \\ V_{33}=a_{33}Y_{10}^{g_{31,33}}Z_{33}, \\ V_{34}=a_{34}Y_{11}^{g_{33,34}}Z_{34}, \\ V_{35}=a_{35}Y_{12}^{g_{32,35}}Z_{35}, \\ V_{36}=a_{36}Y_{13}^{g_{34,36}}Z_{36}, \\ V_{37}=a_{37}Y_{13}^{g_{34,37}}Z_{37}, \\ V_{38}=a_{38}Y_{15}^{g_{6,38}}Z_{38}. \\ \end{matrix} \end{matrix}$ (4)

$\begin{matrix} E_{1}=e_{1}X_{4}^{g_{12,39}}Z_{39}, \\ E_{2}=e_{2}X_{12}^{g_{20,40}}Z_{40}, \\ E_{3}=e_{3}Y_{4}^{g_{2,41}}Z_{41}, \\ E_{4}=e_{4}Y_{12}^{g_{32,42}}Z_{42}, \end{matrix}$ (5)

where $a_{i}$’s and $e_{i}$’s are rate constants, and $Z_{i}$’s are the fluxes’ corresponding enzymes. Diffusion fluxes are modeled as

$\begin{matrix} \begin{matrix} D_{1}=d_{1}\cdot\left(X_{2}-Y_{2}\right), \\ D_{2}=d_{2}\cdot\left(Y_{4}-X_{4}\right), \\ D_{3}=d_{3}\cdot\left(X_{8}-Y_{8}\right), \\ D_{4}=d_{4}\cdot\left(Y_{9}-X_{9}\right), \\ D_{5}=d_{5}\cdot\left(X_{14}-Y_{14}\right), \\ D_{6}=d_{6}\cdot\left(X_{15}-Y_{15}\right), \\ D_{7}=d_{7}\cdot\left(Y_{16}-X_{16}\right), \end{matrix} & & \begin{matrix} D_{8}=d_{8}\cdot\left(Y_{17}-X_{17}\right), \\ D_{9}=d_{9}\cdot\left(Y_{5}-X_{5}\right), \\ D_{10}=d_{10}\cdot\left(Y_{6}-X_{6}\right), \\ D_{11}=d_{11}\cdot\left(Y_{10}-X_{10}\right), \\ D_{12}=d_{12}\cdot\left(Y_{12}-X_{12}\right), \\ D_{13}=d_{13}\cdot\left(Y_{11}-X_{11}\right), \\ D_{14}=d_{14}\cdot\left(Y_{13}-X_{13}\right), \end{matrix} \end{matrix}$ (6)

where $d_{i}$’s are rate constants.
